# Global variations in diagnostic methods and epidemiological estimates in Pompe disease: findings from a scoping review

**DOI:** 10.1186/s13023-025-03679-3

**Published:** 2025-05-06

**Authors:** Roberto Giugliani, Faryn Solomon, Hani Kushlaf, Erica Wright, Tmirah Haselkorn, Edmar Zanoteli, Benedikt Schoser

**Affiliations:** 1 Department of Genetics, UFRGS, Medical Genetics Service, HCPA, INAGEMP, Dasa Genomics, and Casa dos Raros, Porto Alegre, Brazil; 2 Astellas Gene Therapies, San Francisco, CA USA; 3 University of Cincinnati College of Medicine, Cincinnati, OH USA; 4 University of Colorado Anschutz Medical Campus, Aurora, CO USA; 5 University of São Paulo, São Paulo, Brazil; 6 Friedrich-Baur-Institute and Department of Neurology, Ludwig-Maximilians University, Ziemssentr. 1, 0336 Munich, Germany

**Keywords:** Pompe disease, Glycogen storage disease type II, Acid maltase deficiency, Prevalence, Epidemiology

## Abstract

**Background:**

Pompe disease is caused by pathogenic variants in the *GAA* gene, resulting in lysosomal acid α-glucosidase (GAA) deficiency. The prevalence of Pompe disease is not well-defined, and estimates vary by geographic region. We evaluated the global epidemiology of Pompe disease and the potential reasons for differing prevalence estimates using published data from worldwide newborn screening (NBS) programs and population-based studies.

**Methods:**

A comprehensive literature search in PubMed was conducted in July 2023, updated in March 2024, and validated with an Embase search in June 2024. Search terms included Pompe disease, GSDII, prevalence, incidence, epidemiology, survival, mortality, and NBS. Studies were included based on robust epidemiological methods, the presence of disease definition, and publication within the past 5 years. We identified 1210 abstracts, of which 295 met recency criteria, 30 were deemed relevant, and 11 met all inclusion criteria.

**Results:**

Prevalence estimates and GAA enzyme activity cutoff values varied across geographic regions. In NBS studies, the birth prevalence of infantile-onset Pompe disease (IOPD) ranged from 1 in 297,387 in Japan to 1 in 62,186 in Taiwan, and late-onset Pompe disease (LOPD) ranged from 1 in 82,914 in Taiwan to 1 in 17,133 in Pennsylvania. Data from the French National Pompe Registry (N = 246) showed an increase in diagnosis of LOPD from 2.6/year before 2001 to 10.6/year during 2001–2010 and 12.8/year during 2011–2015. Enzyme cutoffs in dried blood spots varied from < 3% of lymphocyte mean to 2.10 μmol/L/h to ≤ 18% of the daily median. Three studies noted higher prevalence in populations of African descent, and two noted a higher frequency of pseudodeficiency alleles in Asian populations.

**Conclusions:**

This scoping review confirmed that prevalence estimates differ for IOPD and LOPD and vary by geographic region, potentially by race and ethnicity. It highlights the need to standardize screening and diagnosis methods, genetic testing protocols, and uniform disease classification between IOPD and LOPD.

## Introduction

Pompe disease (also known as glycogen storage disease type II or acid maltase deficiency) is a multisystemic metabolic disease caused by pathogenic variants in the *GAA* gene that encodes α-alpha-glucosidase (GAA), the enzyme that breaks down glycogen into glucose in lysosomes [1]. The resulting GAA deficiency leads to lysosomal glycogen accumulation in many tissues, primarily skeletal, cardiac, smooth muscle, and the central nervous system (CNS). This results in progressive muscle weakness, cardiomegaly, respiratory dysfunction, and functional disability [1, 2]. The clinical spectrum varies widely concerning age at onset, disease progression rate, and extent of organ involvement [3, 4]. Although Pompe disease phenotypes exist on a continuum [5], the disease is broadly categorized into infantile-onset Pompe disease (IOPD) and late-onset Pompe disease (LOPD). The core presentation of IOPD is rapidly progressive and characterized by prominent cardiomegaly, hepatomegaly, and CNS involvement, leading to weakness, hypotonia, and death due to cardiorespiratory failure within the first year of life; however, milder phenotypes with slower progression and less severe cardiomyopathy do present in the first year of life [6]. LOPD is characterized by slowly progressive vacuolar myopathy predominantly involving skeletal muscle, which leads to respiratory and mobility dysfunction and often the need for respiratory and ambulatory support; typically, there is no cardiac involvement [7]. Symptom onset in LOPD occurs in childhood, generally after 1 year of age, or adulthood [6].

Pompe disease is diagnosed by testing for GAA enzyme deficiency and/or disease-causing variants in the *GAA* gene [6] or via newborn screening (NBS) programs. However, some Pompe genotypes are variants of unknown significance and may be classified as potential or suspected cases [8]. There is no precise genotype–phenotype correlation [7]. Furthermore, patients with pseudodeficiency alleles (i.e., those that alter the protein or change gene expression without causing disease) may be identified by NBS programs if they rely on biochemical GAA enzyme analysis without confirmation by molecular *GAA* analysis. NBS programs screen for and diagnose Pompe disease at birth, but in most cases, testing methods are program-dependent, even in the presence of national NBS recommendations. In regions without NBS programs, low awareness of Pompe disease among healthcare providers and scarce test availability may limit access to diagnostic testing.

Pompe disease's frequency (prevalence or incidence) is not well-defined, and estimates vary by geographic region [9–12]. The highly cited prevalence of 1 in 40,000 originates from two studies published in the 1990s in the Netherlands and New York state (USA) [9, 11]. More recent studies suggest that the prevalence of Pompe disease has increased over time with the addition of Pompe disease NBS panels and the rise of disease awareness among healthcare providers [10, 12, 13]. Estimating the disease frequency of Pompe disease is complicated by several factors, including heterogeneity of presentation and phenotype, differing cut-off values for GAA enzyme activity, absence of standardized diagnostic criteria in NBS programs, lack of uniform disease classification and age cut-offs to distinguish between IOPD and LOPD, and uncertainty about factors that contribute to clusters or regional variation in estimates. Uniform and consistent long-term follow-up of patients with Pompe disease identified by NBS is needed to better understand the phenotype/genotype correlation and accurately provide incidence/prevalence of IOPD and LOPD.

We conducted a scoping review to assess the current literature reporting epidemiologic estimates for Pompe disease and identify limitations of the current data. By understanding these limitations, we aimed to raise awareness and lay the groundwork for more consistent NBS approaches, patient identification, and ultimately, more consistent and reliable estimates for the true prevalence of Pompe disease.

## Methods

We conducted a comprehensive literature search in PubMed in July 2023 on the global epidemiology of Pompe disease. The PubMed search was updated in March 2024 by one reviewer and validated by an independent second reviewer by performing the same search in Embase in June 2024. The search terms were: “Pompe disease”, “glycogen storage disease type II”, “prevalence”, “incidence”, “epidemiology”, “survival”, “mortality”, and “newborn screening.” Results were restricted to English language publications and exported as an Excel file. A formal review protocol was not registered. Studies were selected for inclusion based on the following criteria: epidemiologically sound methods including appropriate base population and calculations; prevalence defined as the proportion of existing cases in a population during a specific point in time or period; incidence defined as new cases in a specified population at a specified time, presented as a function of time or rate; and birth prevalence defined as the proportion of cases diagnosed at birth among the total number of infants screened. One way in which birth prevalence is calculated is through the proportion of cases diagnosed through mandatory NBS programs.

We included studies that defined IOPD and LOPD using a cutoff age of 12 months, whereby symptom onset before age 12 months was classified as IOPD and symptom onset at 12 months or later was classified as LOPD. We included one large, well-designed study that used a 2-year cutoff age [12]. Studies with any GAA cutoff value were included, as consensus for GAA cutoff values is lacking. Since diagnostic methods and Pompe disease awareness have changed over time, we limited our search to publications within 5 years from the search date and excluded pilot studies.

We excluded publications in which no denominator was provided, no epidemiological estimate was provided, the methodology was not clearly described, and the authors explicitly stated a lack of accuracy or reliability regarding patients’ disease status. We also excluded studies for any of the following reasons: prevalence was calculated as existing patients at a given age divided by the number of infants born in the corresponding birth year, or our calculations of the prevalence/incidence did not match the presented prevalence/incidence or an estimate was calculated based on carrier frequencies. All results of this scoping review reported herein are based on the Preferred Reporting Items for Systematic reviews and Meta-Analyses extension for Scoping Reviews (PRISMA-ScR) [14].

## Results

**Fig. 1:**
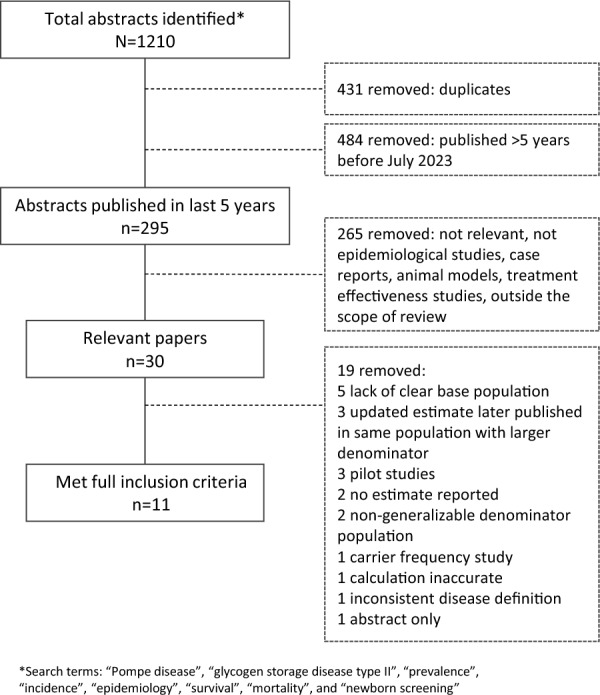
Study selection process

We identified 1210 abstracts, of which 295 met recency criteria, 30 were deemed relevant, and 11 were full publications that met all inclusion criteria (Fig. 1).

Pompe disease was diagnosed by NBS using GAA enzyme assay and confirmation by genetic testing or clinically with confirmation by GAA enzyme assay and, in many cases, confirmation by genetic testing (Table 1). Various enzyme activity cutoffs were used for diagnosis, including < 3% of lymphocyte mean, 2.10 μmol/L/h in dried blood spots (DBS), ≤ 18% of the daily median in DBS, 10% of mean GAA activity in DBS, and < 4.0 pmol h^−1^ disk^−1^ or < 3.5 pmol h^−1^ disk^−1^ in DBS. Four of 11 studies reported using some form of second-tier or third-tier testing, whereby *GAA* full gene sequencing was performed after a second (or third in one study) GAA enzyme activity test was below the cutoff value for both positive and borderline cases or only for borderline cases. Eight studies did not report the use of enzymatic tiered testing.

**Table 1:** Characteristics of studies included in the scoping review

Author, publication year	Region/country	Race/ethnicity distribution	Study type/ methods	Assay type	GAA enzyme activity cut-off value		Genetic Confirmation
					IOPD	LOPD	
Burton, et al. 2020 [10]	Illinois, USA	Not specified	Newborn screening	MS/MS from DBS [43]	Positive ≤ 18% of daily median GAA activity; Borderline > 18% to ≤ 22%		Molecular testing done on all samples below cutoff
Elenga, et al. 2017 [21]	French Guiana	Maroon population/all of African descent)	Nationwide, retrospective records	DBS	Cutoff unknown	–	10 of 19 patients genotyped, 2 partially genotyped
Ficicioglu et al. 2020 [8]	Pennsylvania, USA	Not specified	Newborn screening	Flow-injection tandem mass spectrometry (FIA/MS/MS) from DBS	≈ 18% of the daily mean		All cases genetically confirmed after 2nd tier cutoff
Gragnaniello, et al. 2023 [36]^#^	Northeastern Italy	Diagnosed population: 69.2% European, 12.8% Asian, 10.3% African, 7.7% unknown	Newborn screening	MS/MS [44]	Multiple of the median (MoM) 0.2 in DBS		Confirmatory testing on all
Lee et al. 2022 [16]	Taiwan	Not specified	Newborn screening	Fibroblasts and HEK293T cells	< 3% of normal mean (lymphocyte) with cardiac involvement	< 3% of normal mean (lymphocyte)	Confirmatory testing on samples below cutoff
Limgala, et al. 2021 [17]	Washington, DC, USA	Screened population: 90% African descent, 5% Hispanic, 5% Caucasian	Outpatient cohort; enzyme tested	Fluorometric assays on DBS	–	10% of mean GAA activity	Molecular confirmation on all samples below 2nd tier cutoff
Navarrete-Martínez, et al. 2017 [18]	Mexico	Not specified	Newborn screening	MS/MS on DBS	2.10 μmol/L/h Pompe disease type not specified		Molecular confirmation on all samples below 2nd tier cutoff
Sawada, et al. 2021 [15]	Japan	Not specified	Newborn screening	Fluorometric assay on DBS	DBS cutoff < 4.0 pmol ${\text{h}}^{-1} {\text{disk}}^{-1}$ (method I), < 3.5 pmol ${\text{h}}^{-1} {\text{disk}}^{-1}$ (methods II, III)		Gene sequencing on all samples below 3rd tier cutoff
Semplicini, et al. 2018 [12]	France	Not specified	Nationwide, observational records	Three options: (1) Enzymatic test on lympho/leukocytes and confirmation on fibroblasts, or (2) enzymatic test on lymphocytes/leukocytes and confirmation by *GAA* gene sequencing, or (3) enzymatic testing on DBS and confirmation by *GAA* gene sequencing	–	Cut-off not reported; LOPD defined as ≥ 2 years old at diagnosis	204 of 246 patients genetically sequenced
Tang, et al. 2020 [20]	California, USA	Screened population:* 8.6% AA, 15.9% API, 49% Hispanic, 26% White. Of pathogenic alleles, 11.5% AA, 28.8% API, 26.9% Hispanic, 32.7% White	Newborn screening	FIA-MS/MS on DBS	< 10–18% of the daily median		DNA sequencing on all samples below first cutoff
Theadom, et al. 2019 [19]	New Zealand	70% European, 20% Māori, 10% Asian	Nationwide, population-based case ascertainment	Not specified	–	Pompe disease type not specified	2 of 10 cases molecularly confirmed

*AA* African American, *API* Asia–Pacific Islander, *DBS* dried blood spot, IOPD infantile-onset Pompe disease, *LOPD* late-onset Pompe disease

*Race presented by allele frequency, not by person/case

^#^This paper reports the most recent cumulative data from 8 years of a regional newborn screening program. Two earlier publications reported data at 17 months and 7 years [44, 45]

**Fig. 2:**
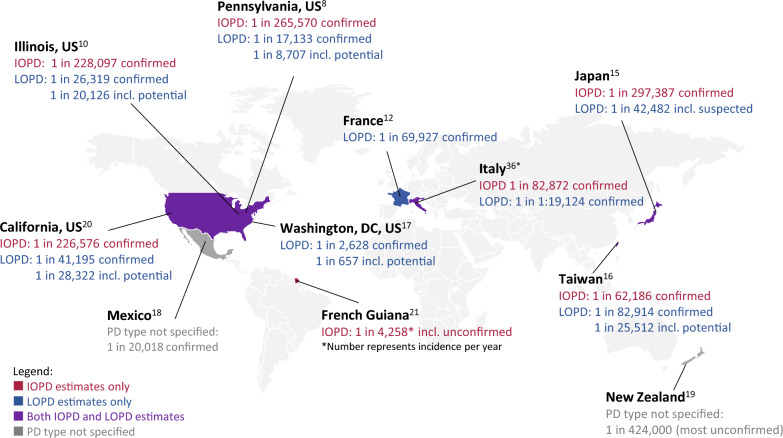
Birth prevalence by country/region for infantile-onset pompe disease (IOPD), late-onset pompe disease (LOPD) and Pompe Disease (PD)—type not specified. *This paper reports the most recent cumulative data from 8 years of a regional newborn screening program. Two earlier publications reported data at 17 months and 7 years [44, 45]

Prevalence estimates for IOPD and LOPD varied widely worldwide (Fig. 2). Genetically confirmed IOPD birth prevalence ranged from 1 in 297,387 in Japan [15] to 1 in 62,186 in Taiwan [16]. Genetically confirmed LOPD birth prevalence ranged from 1 in 82,914 in Taiwan [16] to 1 in 17,133 in the US state of Pennsylvania [8]. When unconfirmed LOPD cases were included (i.e., potential/suspected Pompe disease), the prevalence of LOPD ranged from 1 in 42,842 in Japan [15] to 1 in 657 in Washington, DC, USA [17]. The analysis from the French National Pompe Registry showed an increase in diagnosis of LOPD (defined as symptom onset at or after 2 years of age) from 2.6/year before 2001 to 10.6/year during 2001–2010 and 12.8/year during 2011–2015 [12]. Two studies reported the prevalence of Pompe disease, without specifying IOPD or LOPD, as 1 in 20,018 (genetically confirmed) in Mexico [18], and 1 in 424,000 (mostly unconfirmed) in New Zealand [19].

Some of these studies examined prevalence according to sex, race or ethnicity. Studies that reported data separately for males and females showed that Pompe disease affects males and females equally [12, 17, 20]. Three studies noted higher prevalence in populations of African descent. The first-year experience of NBS for Pompe disease in California reported a prevalence of 1 in 18,700 African-American newborns relative to an overall birth prevalence of 1 in 25,175 [20]. A study in Washington, DC, in a population consisting of 90% African Americans, reported a LOPD prevalence of 1 in 2628 confirmed cases [17]. A retrospective records review in the Maroon community of French Guiana, a population of African descent, reported an IOPD incidence of 1 in 4258 without genetic confirmation [21]. The authors of this last study noted that this relatively small population has a reduced pool of genotypes due to seldom mixing with other populations, and only two pathogenic variants were present, which is compatible with a double founder effect [21]. Two studies noted a higher prevalence of pseudodeficiency alleles in Asian populations. In Taiwan, 40% of Pompe disease patients detected by NBS carried the pseudodeficiency variant c.[1726G > A;2065G > A] that represents 80% of all pseudodeficiency alleles [16]. In the California NBS program, there was a high frequency of pseudodeficiency alleles (1 in 2300) among ethnic Asia/Pacific Islander newborns, especially the c.[1726G > A;2065G > A] variant [20].

## Discussion

First-generation enzyme replacement therapy for Pompe disease was approved in the United States for treating IOPD in 2006 and LOPD in 2010 [22, 23], and is now available in more than 65 countries. With the widespread availability of this disease-modifying treatment, establishing reliable estimates of Pompe disease prevalence is essential for ensuring that patients are diagnosed and treated promptly. In this scoping review of primary data from NBS programs and population-based studies within the last 5 years, prevalence estimates for IOPD and LOPD varied widely across the eight represented countries.

Until recently, there were no large-scale analyses of global Pompe disease prevalence. In January 2024, an analysis of NBS data collected from 22 US states and eight countries on four continents estimated birth prevalence of Pompe disease at 1 in 18,711 overall (IOPD 1 in 126,118 and LOPD 1 in 21,902) [13]. Diagnosis of Pompe disease was based on low GAA enzyme activity and two (or more) identified pathogenic or likely pathogenic *GAA* variants. That study, in which the varied estimates from large and small populations were pooled for analysis, did not find differences in prevalence across populations of European, Latin American, or Asian ancestry. This contrasts with substantial evidence of geographic variation from studies using other methods to calculate prevalence [8–12, 20, 24–34]. Our review of prevalence estimates published within the last 5 years did point to higher disease prevalence among populations of African descent [17, 20, 21] and pseudodeficiency alleles among Asian populations [16, 20].

Combining data from NBS programs worldwide to develop a global prevalence estimate of Pompe disease is appealing. However, significant differences across these disparate datasets should be considered. NBS screening programs are not standardized across countries or even from one US state to another. The cut-off values for diagnosing Pompe disease also differ depending on the type of enzyme activity assay (e.g., lymphocyte assay, digital microfluidics, or tandem mass spectrometry) and from one laboratory to another. Cut-off values and assay type affect how many patients test positive for Pompe disease on an enzyme assay and move on to higher-tier testing or genetic testing to confirm the diagnosis. When positive cases are missed in the first round of enzyme assay testing, these patients may go undiagnosed for years until symptom development leads to an eventual diagnosis. On the other hand, higher cut-off values without genetic confirmation increase the chance of patients with pseudodeficiency alleles (common in Taiwan and the United States) erroneously being diagnosed with Pompe disease and skewing estimates. Thus, prevalence estimates need to be reported in the context of the different cut-off values, type of assay, and pick-up rates for DBS (e.g., in one of the largest screened LOPD cohorts, the DBS prevalence of LOPD is reported at 2.4% [35]). The prevalence of Pompe disease would be best determined by genetic testing following a DBS protocol. Furthermore, an international standard for calculating prevalence is also needed. For example, calculating birth prevalence as the number of adult Pompe patients divided by the number of newborns in their respective birth years would underestimate prevalence. In addition, the small sample sizes common in rare diseases leave these studies susceptible to cluster or founder effects that may not represent the broader disease population.

Several additional factors can influence prevalence estimates, including case definitions (e.g., definite, probable, or possible Pompe disease), assay type (tandem mass spectrometry or fluorometry), confirmation methods (biochemical and/or genetic) and variants of uncertain significance. The technique used for NBS should always be specified because the analytical range of mass spectrometry is higher than that of fluorometric assays. This allows for more accurate enzyme activity measurement at very low values, potentially improving differentiation between patients with pathogenic variants, pseudodeficiency alleles and/or benign variants [36]. The year in which birth prevalence rates are reported is also essential, as each state/country implemented NBS at different times, and there are likely differences in birth prevalence between pre- and post-implementation of NBS.

The different phenotypes and disease courses in IOPD and LOPD, and the fact that treatments are studied independently in these two populations, justify calculating prevalence separately. The current classification is based on age of onset: IOPD for onset before age 1 year and LOPD for onset after age 1 year or onset before age 1 year without cardiomyopathy [31]. Admittedly, the disease phenotype exists on a spectrum, and this binary classification is imperfect. Further studies based on consistent, standardized follow-up of patients diagnosed via NBS are needed to better differentiate between IOPD and LOPD and clarify phenotype-genotype correlations.

Screening for Pompe disease is not mandatory worldwide [13]. In the USA, Pompe disease was added to the Recommended Uniform Screening Panel in 2015, and most states (all besides Texas and Montana) have now adopted it into their NBS programs, albeit at varying rates [13]. NBS for treatable, severe congenital disorders can identify affected infants before the onset of life-threatening manifestations, particularly for patients with limited access to diagnostic services, detect specific populations at increased risk, and ultimately allow for optimized treatment. For example, population analysis of NBS for biotinidase deficiency in California identified an unexpectedly high incidence of profound deficiency, approximately twice the incidence reported worldwide, driven by a higher incidence of profound deficiency among Hispanic newborns [37]. Similarly, mandatory NBS for severe combined immunodeficiency (SCID) in the Navajo Nation population, where there is increased frequency due to a founder mutation, detects otherwise unsuspected SCID diagnoses [38]. Implementing routine NBS for spinal muscular atrophy (SMA) has helped expand the benefits of early treatment with the recent availability of novel therapies for SMA [39]. NBS in SMA results in presymptomatic treatment and improves outcomes in children with genetically proven SMA. Nevertheless, NBS in SMA has a psychosocial impact on families, not only in terms of diagnosis but especially in terms of treatment, and triggers concerns about the future, emphasizing the need for comprehensive multidisciplinary care. Understanding the parents' perspective allows disease specialists and genetic counselors to develop a care plan for parents during the challenging time of uncertainty, anxiety, frustration, and fear of the unknown [40–42]. These aspects also need to be considered in the case of Pompe disease.

Scoping reviews have inherent limitations, as they are intended to qualitatively summarize existing literature and identify gaps in research or practice. Although we applied methodologic criteria for calculating prevalence estimates in each study, we did not evaluate the selected studies according to a level of evidence hierarchy or risk of bias. By limiting our review to the most recent 5 years, we provide data that reflect the current landscape with detection by NBS. Still, we excluded earlier, much-cited prevalence estimates prior to the NBS era.

In summary, this scoping review showed that prevalence estimates differ for IOPD and LOPD and vary widely by geographic region, potentially by race and ethnicity. Most importantly, it highlights the need for uniformity in diagnostic and screening methods, disease classification, counselling, and family care. Continued efforts are needed to refine and standardize screening and diagnosis methods, genetic testing protocols, and the differentiation of IOPD from LOPD. Prevalence estimates need to be reported, including information on the type of assay, cutoff values, DBS pick-up rates, and confirmatory genetic testing. A repository of NBS results is required to collect long-term data and analyze the genotypic-phenotypic spectrum of Pompe disease.

## Data Availability

Not applicable. There is no proprietary data set to share. The data were collected from published studies.
